# Supplementary material for: The CRK5 and WRKY53 Are Conditional Regulators of Senescence and Stomatal Conductance in Arabidopsis
Source: Cells. 2022 Nov 10;11(22):3558. doi: 10.3390/cells11223558 (PMC9688832; doi:10.3390/cells11223558)
Supplement: Supplementary file 1 [file cells-11-03558-s001.zip › cells-2012687-supplementary.pdf]

Supplementary data

Table S1. List of all proteins identified as CRK5 interactors during yeast two hybrid cDNA library screening. The analysis was performed by yeast two-hybrid screening using Mate & PlateTM Library. Screening identified 32 interacting proteins. The transformed colonies representing the appropriate proteins were positively screened for growth on quadruple selection SD medium lacking adenine, leucine, histidine and tryptophan with the addition of aureobasidin A. The "score" section represents the number of different yeast colonies carrying the appropriate gene identified during the experiment.

	Locus	Name/Description	Score
1.	AT5G66680	DEFECTIVE GLYCOSYLATION, DGL1, dolichyl-diphosphooligosaccharide-protein glycosyltransferase 48kDa subunit	1
2.	AT5G47100	CALCINEURIN B-LIKE PROTEIN 9	2
3.	AT5G20740	Plant invertase/pectin methylesterase inhibitor superfamily protein	2
4.	AT5G59880	ACTIN DEPOLYMERIZING FACTOR 3, ADF3	9
5.	AT4G02580	NADH-ubiquinone oxidoreductase 24 kDa subunit	1
6.	AT5G40170	RECEPTOR LIKE PROTEIN 54	1
7.	AT5G17860	CALCIUM EXCHANGER 7, Cation/Ca2+ exchanger family member	1
8.	AT5G11790	N-MYC DOWNREGULATED-LIKE 2, NDL2	1
9.	AT3G52760	Integral membrane Yip1 family protein	1
10.	AT3G14080	Small nuclear ribonucleoprotein family protein	4
11.	AT4G08930	protein disulfide isomerase APR-like 6	2
12.	AT1G07140	Ran-binding protein (siRanBP)	1
13.	AT1G06890	nodulin MtN21 /EamA-like transporter family protein	1
14.	AT1G56300	Chaperone DnaJ-domain superfamily protein	1
15.	AT1G67360	Rubber elongation factor protein (REF)	1
16.	AT1G76540	cyclin-dependent kinase B2	1
17.	AT4G17510	ubiquitin C-terminal hydrolase 3	1
18.	AT2G27180	hypothetical protein	1
19.	AT5G67480	BTB and TAZ domain protein 4 (BT4)	1
20.	AT5G62360	Plant invertase/pectin methylesterase inhibitor superfamily protein	1
21.	AT1G72190	D-isomer specific 2-hydroxyacid dehydrogenase family protein	1
22.	AT1G10657	Plant protein 1589 of unknown function	1
23.	AT3G12345	FKBP-type peptidyl-prolyl cis-trans isomerase	1
24.	AT1G10610	basic helix-loop-helix (bHLH) DNA-binding superfamily protein	1
25.	AT4G14130	xyloglucan endotransglucosylase/hydrolase 15	1
26.	AT2G33250	transmembrane protein	1
27.	AT1G51160	SNARE-like superfamily protein	1
28.	AT3G45280	syntaxin of plants 72	1
29.	AT4G26080	ABA INSENSITIVE 1 protein phosphatase 2C	1
30.	AT1G19330	Histone deacetylase complex subunit SAP30	1
31.	AT1G26650	Son of sevenless protein	1
32.	AT1G48100	POLYGALACTURONASE INVOLVED IN EXPANSION 3, Pectin lyase-like superfamily protein	1

Table S2. List of CRK5-interacting proteins associated with water deficit, calcium signalling or cell wall composition.

	Locus	Name	GO – biological process	References
1.	At5g47100	CALCINEURIN B-LIKE PROTEIN 9, CBL9	response to water deprivation, stomatal movement, abscisic acid-activated signaling pathway	Drerup et al., 2013; Gao and Zhang, 2019
2.	At5g17860	CALCIUM EXCHANGER 7, CAX7	cation transmembrane transport	Li et al., 2016
3.	At3g14080	Sm-like protein LSM1B	hyperosmotic salinity response, response to cold and water deprivation	Perea-Resa et al., 2016
4.	AT4G26080	ABA INSENSITIVE 1, ABI1	negative regulation of abscisic acid-activated signaling pathway, regulation of stomatal movement	Gosti et al., 1999; Krzywinska et al., 2016
5.	AT1G67360	LD-ASSOCIATED PROTEIN 1, LDAP1	positive regulation of response to water deprivation, cellular response to hypoxia,	Kim et al., 2016
6.	AT5g67480	BTB and TAZ domain-containing protein 4, BT4	response to salt stress, wounding, hydrogen peroxide	Du and Poovaiah, 2004
7.	AT1G48100	POLYGALACTURONASE INVOLVED IN EXPANSION3, PGX3	plant-type cell wall modification involved in multidimensional cell growth	Rui et al., 2017
8.	AT5G62360	PECTIN METHYL-ESTERASE INHIBITOR 13, PMEI13	plant-type cell wall modification	Chen et al., 2018
9.	AT4G14130	XYLOGLUCAN ENDOTRANSGLUCOSYLASE/HYDROLASE 15, XTH15	cell wall biogenesis and organization, xyloglucan metabolic process	Rose et al., 2002

Table S3. Primers used in this study

Gene name	AGI code	Sequence LP primer	Sequence RP primer	Used for
CRK5	AT4G23130	AGGAGATCTCTCGCCAGAATC	CGATAGTCTCTTCACGGCAAC	genotyping
WRKY53	AT4G23810	TCAGGCACGACTTAGAGAAGC	GGGAAAGTTGTGTCAATCTCG	genotyping
UPL7	AT3G53090	TTCAAATACTTGCAGCCAACCTT	CAAAGAGAGGTATCACAAGAGACT	qPCR reference
PP2AA3	AT1G13320	TAACGTGGCCAAAATGATGC	GTTCTCCACAACCGCTTGGT	qPCR reference
WRKY53	AT4G23810	CGGAAGTCCGAGAAGTGAAG	GCCTCTCTCTGGGCTTATTC	qPCR
CRK5	AT4G23130	TTGTTGTGCCAGTCGCTATCTCAGT	ACCCTGCAGTTGTGATGTCATCCTC	qPCR
CRK5	AT4G23130	CACCGATTTGAGTTTCACATCATAG	TATCCAATTTCTTCACCTTTCT	amplification of *CRK5* promoter
CRK5	AT4G23130	CACCATGTCTGCTTATACCTCATTA	ACGAGGAGCTAAAATAGTAATC	amplification of *CRK5* coding sequence
HMG14	AT2G34450	CACCATGACGAAGAGAGCTCCCAA	TTCAGAATAGTCTGAGTCGGTC	amplification of *HMG14* coding sequence
WRKY6	AT1G62300	CACCATGGACAGAGGATGGTCTGG	TTGATTTTTGTTGTTTCCTTCG	amplification of *WRKY6* coding sequence
WRKY22	AT4G01250	CACCATGGCCGACGATTGGGATCT	TATTCCTCCGGTGGTAGTGGCG	amplification of *WRKY22* coding sequence
WRKY30	AT5G24110	CACCATGGAGAAGAACCATAGTAG	AGAATAGAACCCACCAAATCC	amplification of *WRKY30* coding sequence
WRKY53	AT4G23810	CACCATGGAAGGAAGAGATATGTT	ATAATAAATCGACTCGTGTAAA	amplification of *WRKY53* coding sequence
WRKY54	AT2G40750	CACCATGGATTCGAATAGTAACAA	CATAGCACTTGTTCTTTCATAA	amplification of *WRKY54* coding sequence
WRKY70	AT3G56400	CACCATGGATACTAATAAAGCAAA	AGATAGATTCGAACATGAACTG	amplification of *WRKY70* coding sequence

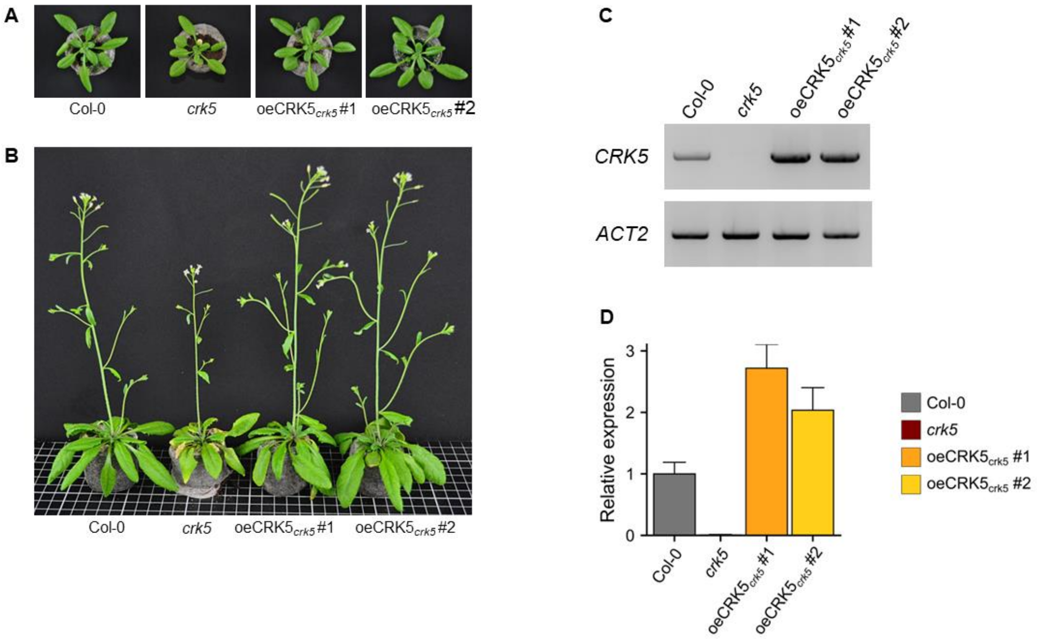

Figure S1: Phenotypic and genetic characterization of plants with altered CRK5 expression used in the study (A) Morphological phenotype of 4-week-old wild type, crk5 and two complementation lines with constitutive expression of CRK5 in the mutant background oeCRK5crk5 #1 and oeCRK5crk5 #2, (B) Morphological phenotype of 6-week-old plants. (C) Quantification of CRK5 transcript in analyzed genotypes using qPCR. (D) qRT-PCR analysis of expression of WRKY53 and CRK5 in the analyzed genotypes. Data show relative expression normalized to the wild type and represent average values ± SD.

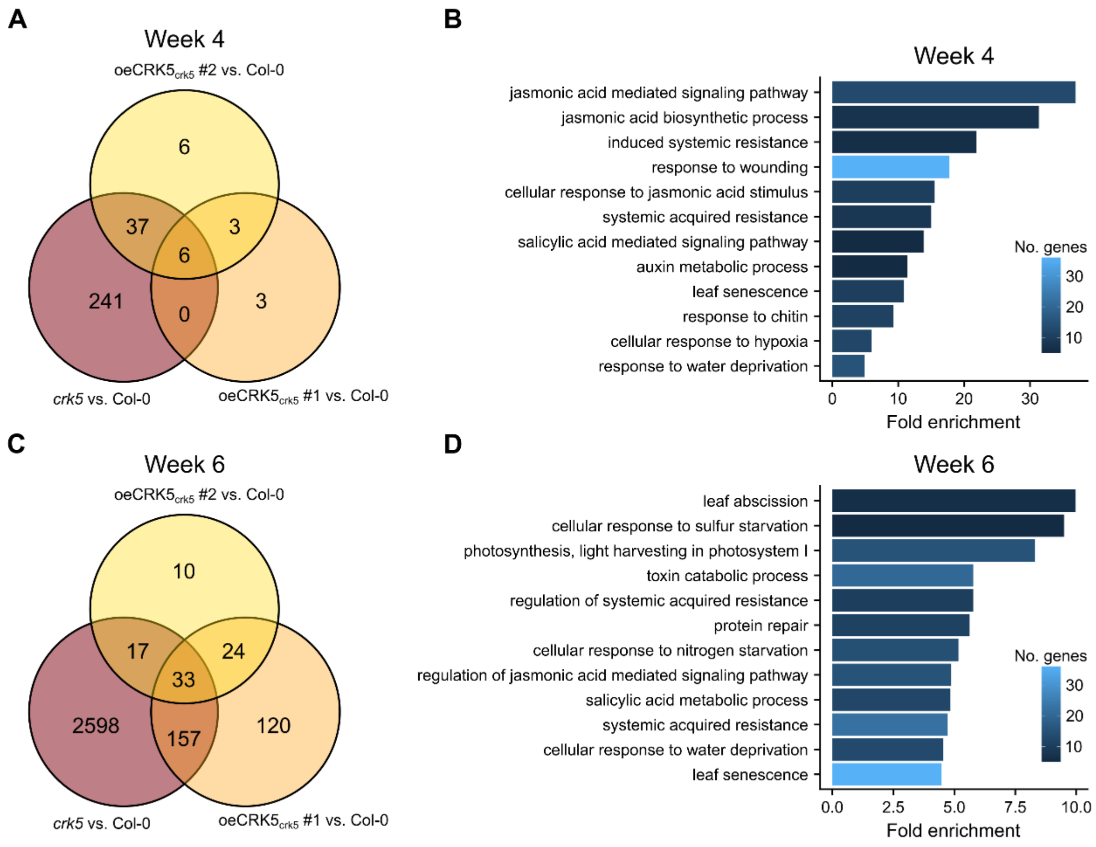

Figure S2: Venn diagram representing differentially expressed genes in 4-week old (A) and 6-week old (C) plants. The study was supported by gene ontology (GO) analysis of genes significantly induced in *crk5* compared to Col-0 in the RNA-seq experiment. Twelve most significantly overrepresented GO terms in 4-week old (B) and 6-week old (D) plants for are presented. The number of genes in each group is shown in color of the bar.

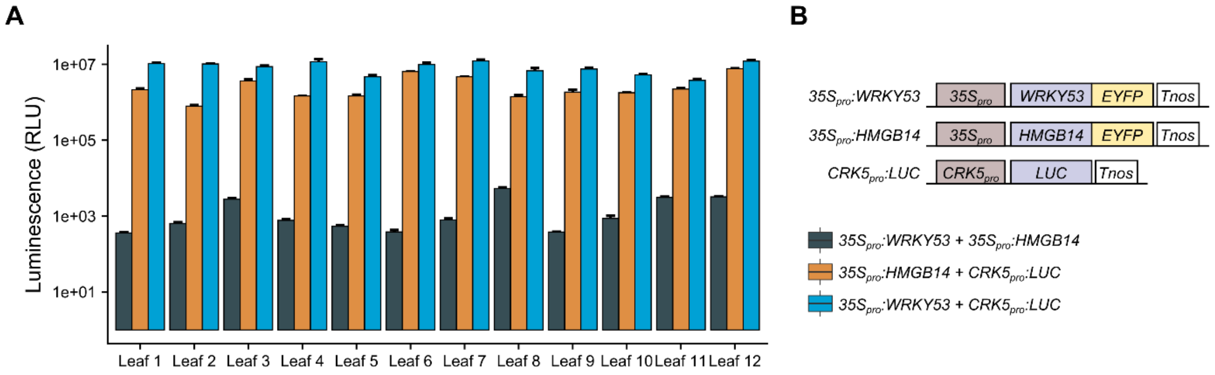

Figure S3: Transient transactivation luciferase reporter assay. The study was based on transient transformation of *N.benthamiana* leaves with two genetic constructs simultaneously: 1) prom35S-WRKY53 and prom35S-HMG At2g34450 (negative control without luciferase activity), 2) prom35S-HMG promCRK5-LUC, 3) prom35S-WRKY53 and promCRK5-LUC. Luciferase activity was quantified in 12 independent tobacco plants. Log scales were used in the graph.

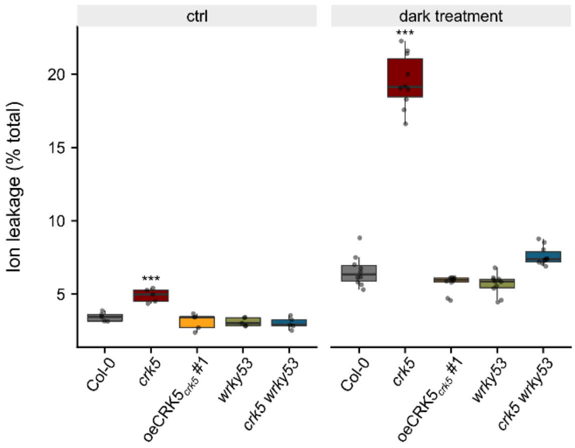

Figure S4: Relative electrolyte leakage in plants after 4-days dark treatment compared to non-treated plants. Mean values (±SD) are derived from 10 independent 4-week old plants (n = 10). Asterisks indicate a significant difference relative to Col-0 according to the one-way ANOVA and Tukey HSD test at level P < 0.001 (***).

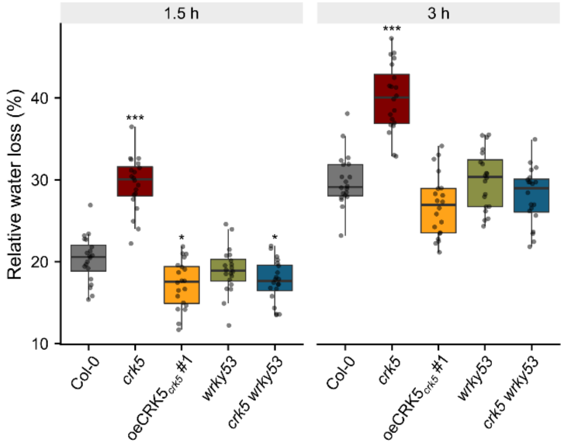

Figure S5: Water losses of detached leaves from analysed genotypes. Water loss was expressed as the percent of the initial fresh weight. Mean values (±SD) were derived from 20 plants (n = 20). Asterisks indicate a significant difference relative to Col-0 according to the one-way ANOVA and Tukey HSD test at level P < 0.001 (***), P < 0.05 (*).

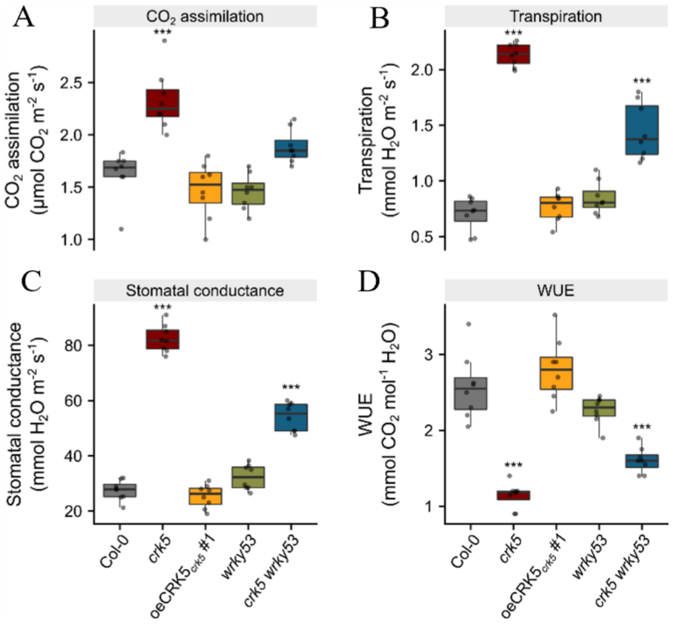

Figure S6: Foliar gas exchange characteristics in plants treated with 450mM NaCl for 7 days. under 400 umol. The analysis was performed on CIRAS-3 Portable Photosynthesis System, with PAR of 300 µmol m-2 s-1, Cuvette Flow of 300 ml/min and intercellular CO_2 concentration of 400 ppm (A/Ci C3 ramp program). Individual charts represent: (A) stomatal conductance, (B) CO2 assimilation, (C) evapotranspiration and (D) water use efficiency (WUE). Mean values (±SD) were derived from 8 plants (n = 8). Asterisks indicate a significant difference relative to Col-0 according to the one-way ANOVA and Tukey HSD test at level P < 0.001 (***).

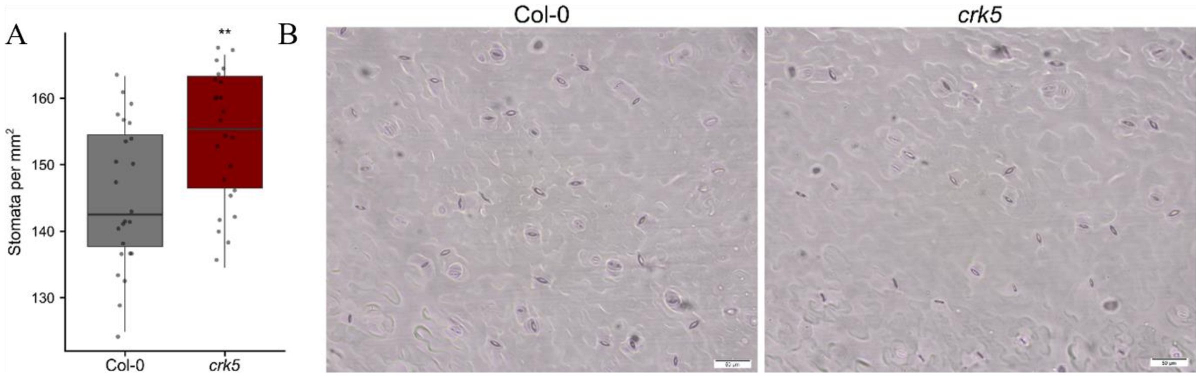

Figure S7: Stomatal aperture in 4-week old plants. (A) Stomatal number calculated per 1mm^2. Mean values (±SD) were derived from the leaves of eight different plants. For each leaf, stomata were counted from three randomly chosen 0.312mm2 picture areas. Asterisks indicate a significant difference relative to Col-0 according to the Students t-test at level P < 0.01 (**). (B) Microscopic pictures taken as an example illustrating stomatal density in Col-0 and *crk5* plants. White scale bars indicate 50 µm.

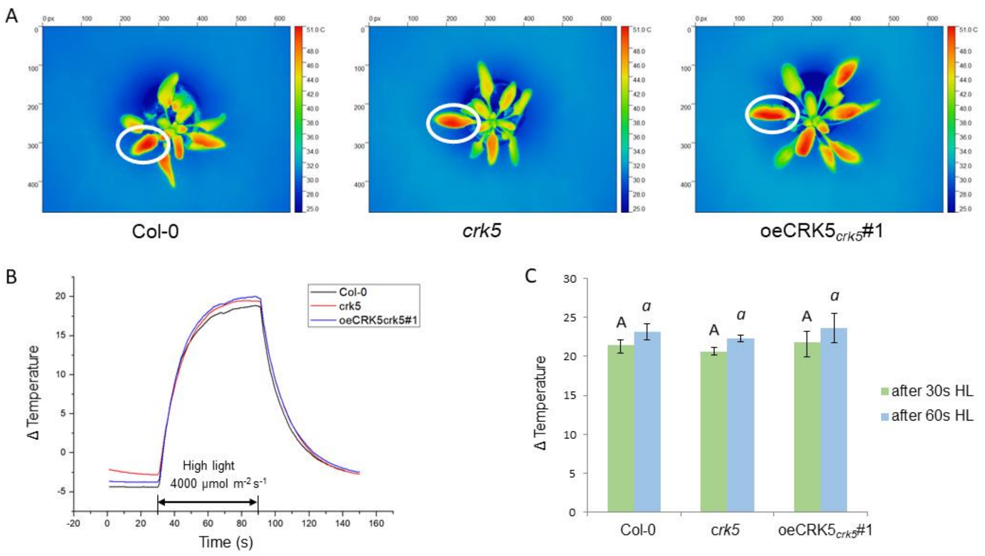

Figure S8: The effect of blocked gas exchange on foliar temperature under variable light conditions. Lanoline was used to reduce leaf water transpiration. (A) Thermograms showing whole rosettes of different *Arabidopsis* genotypes after 30s high light (4,000 µmol photons m^{-2} s^{-1}). Lanoline treated leaves were marked with white circles. (B) Plot showing dynamic temperature changes under variable light conditions. The program was set as follows: 30s of ambient light (150 µmol photons m^{-2} s^{-1}), followed by 60s of high blue light (4,000 µmol photons m^{-2} s^{-1}), followed by 60s of ambient light (150 µmol photons m^{-2} s^{-1}). (C) Average leaf temperature changes after 30s and 60s of high light exposure. Data represent mean values of 4 different leaves from 5 plants (n=20). Statistical analysis was performed according to t-test at level $P < 0.05$.

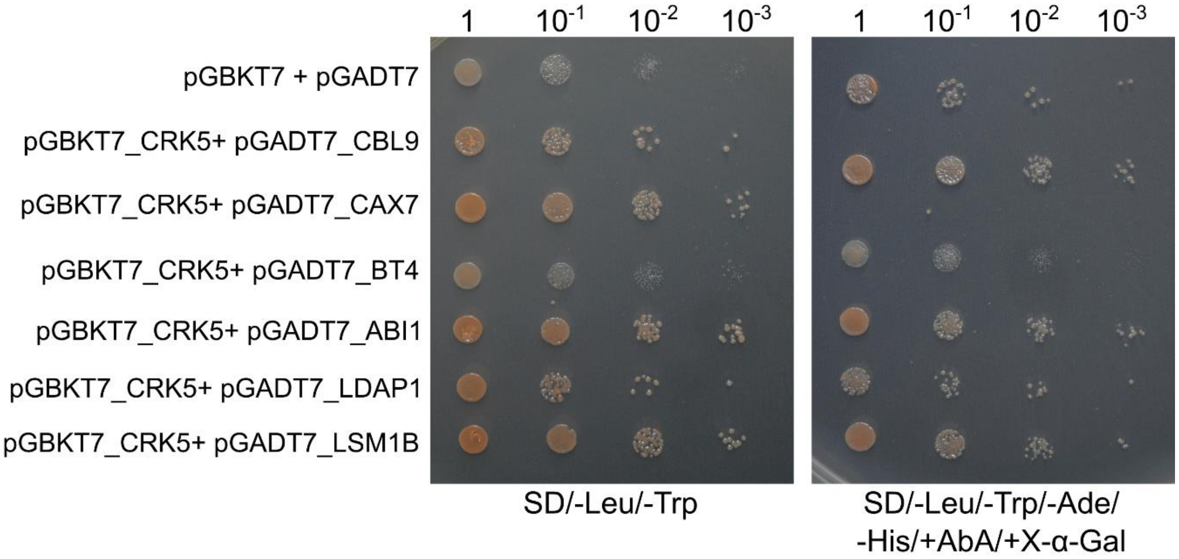

Figure S9: Identification of the interaction between CRK5 and six proteins involved in dehydratation or calcium signaling (CBL9 (At5g47100), CAX7 (At5g17860), BT4 (AT5G67480), ABI1 (AT4G26080), LDAP1 (AT1G67360), LSM1B (At3g14080)). The analysis was performed by Matchmaker Gold Yeast Two-Hybrid System screening. The pGBKT7-CRK5 was screened using Mate & Plate™ Library. Double-deficiency (SD/-Leu/-Trp) screening assay was performed to estimate yeast transformation efficiency, while quadruple dropout medium: SD/–Ade/–His/–Leu/–Trp supplemented with 200 ng/ml aureobasidin was used to identify potential binding partners. Co-transformation with pGADT7 and pGBKT7 was used as a negative control.

Table S4. Statistical analysis of Figure 7c.

		p-values of Tukey HSD test (compared to Col-0)				
time	treatment	crk5	oeCRK5crk5 #1	wrky53	crk5 wrky53	
0	ctrl	9,97E-01	8,55E-01	2,84E-01	9,63E-01	Fv/Fm
2	ctrl	7,47E-01	8,93E-01	9,98E-01	8,93E-01	
4	ctrl	1,00E+00	9,70E-01	9,98E-01	8,81E-01	
7	ctrl	9,99E-01	9,82E-01	8,11E-01	9,99E-01	
9	ctrl	9,87E-01	2,92E-01	9,45E-01	9,99E-01	
11	ctrl	9,99E-01	9,81E-01	9,99E-01	1,00E+00	
14	ctrl	9,99E-01	9,99E-01	9,99E-01	9,88E-01	
0	salt	9,98E-01	1,00E+00	9,70E-01	9,98E-01	
2	salt	9,99E-01	8,50E-01	7,16E-01	9,99E-01	
4	salt	5,94E-03	9,94E-01	2,71E-01	1,05E-02	
7	salt	1,23E-06	9,27E-01	9,27E-01	3,76E-01	
9	salt	1,70E-11	9,71E-01	8,83E-01	2,96E-03	
11	salt	5,60E-08	5,48E-01	1,00E+00	3,97E-05	
14	salt	3,99E-11	3,15E-01	9,90E-01	2,09E-06	
0	ctrl	3,34E-01	3,83E-13	5,16E-01	9,96E-01	Area
2	ctrl	2,82E-03	2,62E-13	7,51E-01	9,68E-01	
4	ctrl	1,90E-01	5,76E-12	9,99E-01	9,62E-01	
7	ctrl	4,96E-03	1,22E-11	7,60E-01	9,27E-01	
9	ctrl	1,20E-02	1,74E-12	4,78E-01	9,73E-01	
11	ctrl	1,82E-02	7,21E-12	6,73E-01	9,88E-01	
14	ctrl	1,20E-02	5,96E-08	9,11E-01	9,88E-01	
0	salt	1,23E-02	4,64E-13	9,96E-01	9,78E-01	
2	salt	5,86E-07	2,49E-13	1,00E+00	5,89E-02	
4	salt	7,06E-08	1,60E-12	9,88E-01	2,20E-03	
7	salt	6,74E-13	2,43E-13	7,82E-01	6,97E-09	
9	salt	2,83E-13	2,40E-13	7,74E-01	5,76E-10	
11	salt	2,43E-13	2,40E-13	9,06E-02	1,84E-12	
14	salt	2,41E-13	2,40E-13	1,17E-01	4,58E-13	
